# Efficacy of tolvaptan in postoperative volume therapy for acute Stanford type A aortic dissection

**DOI:** 10.1186/s12872-023-03125-x

**Published:** 2023-02-21

**Authors:** Wenjun Wang, Feng Gao, Xuezhi He, Yang Gao, Lei Shi, Wei Liu, Xijing Zhuang

**Affiliations:** Department of Cardiovascular Surgery, Dalian Municipal Central Hospital, Dalian, China

**Keywords:** Loop diuretic, Cardiopulmonary bypass, Deep hypothermic circulatory arrest, Replacement of aorta

## Abstract

**Background:**

Despite the increasing application of tolvaptan in cardiac surgery, there is no information on the use of tolvaptan in Stanford patients with type A aortic dissection. This study aimed to evaluate the postoperative clinical effects of tolvaptan in patients with type A aortic dissection  after tafter surgery.

**Methods:**

A retrospective analysis was performed on 45 patients treated for type A aortic dissection in our hospital from 2018 to 2020. These included 21 patients who were treated with tolvaptan (Group T) and 24 patients who received traditional diuretics (Group L). The hospital's electronic health records were used to obtain perioperative data.

**Results:**

Group T did not differ significantly from Group L in terms of the duration of mechanical ventilation, postoperative blood required, length of catecholamine use, or the amount of intravenous diuretic drugs administered (all *P* > 0.05). The development of postoperative atrial fibrillation was significantly less in the tolvaptan group (*P* = 0.023). The urine volumes and change in body weight loss were slightly higher in group T than in group L but the differences were non-significant (*P* > 0.05). Serum potassium, creatinine, and urea nitrogen levels did not differ between the groups in the week after surgery, At the same time, sodium was significantly higher in the Group T group on day 7 after transfer from the ICU (*P* = 0.001). In Group L, sodium levels were also elevated by day 7 (*P* = 0.001). On days 3 and 7, serum creatinine and urea nitrogen levels increased in both groups (both *P* < 0.05).

**Conclusions:**

Both tolvaptan and traditional diuretics were found to be effective and safe for patients with acute Stanford type A aortic dissection. Moreover, tolvaptan may be associated with reducing the incidence of postoperative atrial fibrillation.

## Background

Heart failure may develop during acute type A aortic dissection (AD) due to the necessity for heart arrest during surgery [1–5]. Together with hemodilution, hypothermia, and inflammatory responses, cardiopulmonary bypass and deep hypothermic circulatory arrest (DHCA) can lead to complications such as increased vascular permeability and decreased colloidal osmotic pressure, which can result in interstitial edema [6–9]. Therefore, sufficient volume is required to avert intravascular hypovolemia soon after surgery [10]. The volume is then reduced after stabilizing of inflammation and temperature [11].

The control of excess fluid with diuretics is crucial for the postoperative management of surgery involving the great vessels [12]. Intermittent diuretic and fluid administration is often used during the early postoperative period [14]. Tolvaptan is a vasopressin-2 receptor antagonist that differs in its diuretic mechanism from traditional diuretics, such as furosemide and spironolactone. It acts on distal nephritis and can augment the urine volume without electrolyte removal [13–15]. It has been shown that, in contrast to other diuretics, tolvaptan does not adversely affect blood pressure, renal function, or electrolyte balance [16, 17]. To date, numerous reports have indicated the usefulness of tolvaptan in managing postoperative fluid volumes in patients with cardiac diseases treated with surgery [10].

However, despite the acceptance of tolvaptan for various types of cardiovascular conditions treated by surgery, there is limited information on its use after surgery for type A AD. Therefore, we retrospectively analyzed the clinical effectiveness of tolvaptan in patients after surgery for type A AD in our hospital over two-years, compared with those receiving loop diuretics during the same period.

## Methods

### Study design and ethics approval

There was no precise protocol because this was a retrospective study. The study primarily focused on patient data collected during hospitalization. The Dalian Municipal Central Hospital Ethics Committee approved the study with (Approval No. YN2022-010-01), and the need for informed consent was waived.

### Patients

Fifty-one patients with acute Stanford type A AD were surgically treated at our hospital between January 1, 2018, and December 31, 2020. Four of these patients died after surgery, and two were excluded from the study due to incomplete clinical data.

Of the remaining 45 patients, 21 received tolvaptan after surgery (Group T), while 24 patients did not (Group L).

The normal procedure for cardiac surgery in our hospital is that that patients return to the ward from the intensive care unit (ICU) when their pain VAS score is below 5 and their blood oxygen saturation is continuously higher than 90%.

All patients started oral diuretic therapy (torasemide 20 mg/day oral) on the first day after transfer to the department of cardiac surgery from the ICU and continued for at least one week. The patients receiving tolvaptan were given oral tolvaptan in addition to oral torasemide. Tolvaptan was administered at a 15 mg/day dose for at least one week. Intravenous injection of furosemide (20 mg/potion) or torasemide (10 mg/potion) was used intermittently according to the patient's urine volume.

### Data source and variables

Data were obtained from the hospital’s medical records. The general information including age, sex, body surface area (BSA), body mass index (BMI), comorbidities, and AD etiology, as well as serum levels of alanine transaminase (ALT), albumin, creatinine, and urea nitrogen, were taken at the time of hospital admission.

Intraoperative information included the operative method, operative time, extracorporeal circulation time, and the number and times of DHCAs. Postoperative information of patients included the time spent in ICU, mechanical ventilation time, duration of vasoactive drug use, postoperative blood transfusion volume, length of postoperative hospitalization, the incidence of complications, urine volume, changes of body weight loss, changes in serum sodium, changes in serum potassium, and changes in creatinine and urea nitrogen.

Complications included gastrointestinal bleeding, cerebral infarction, postoperative atrial fibrillation (POAF), and intracranial hemorrhage.

Urine volume: The cumulative urine volume of patients on days 1, 3, and 7 after transfer from the ICU to the cardiac and vascular surgery ward was recorded.

Changes in body weight loss: Patients' cumulative body weight loss on days 1, 3, and 7 after transfer from the ICU to the cardiac and vascular surgery ward were recorded.

Serum sodium, potassium, urea nitrogen, and creatinine: these were determined on days 1, 3, and 7 after transfer from the ICU to the ward.

### Statistical analysis

All data was maintained in Office 365. SPSS Version 26.0 (IBM Corp., Armonk, NY, USA) was used to analyze the data. Data from all measurements are expressed as means ± standard deviation. Student’s t-test was used to assess differences in normally distributed data, while the Wilcoxon rank-sum test was applied for non-normally distributed data. Count data were expressed as the number of cases or composition ratio, and comparisons were performed using the χ^2^ test. Continuous correction χ^2^ tests were utilized if the expected frequency of a cell was greater than 1 and less than 5. Fisher's exact test was employed for measurement data where the predicted frequency of any cell was less than 1. The Wilcoxon rank-sum test was used to compare the test results of the same group of patients at different periods. All the tests were two-tailed, and P-values less than 0.05 (*P* < 0.05) were considered statistically significant.

## Results

### General information

All patients were classified as Penn class Aa. None of the patients had a history of heart failure. None of the patients had a history of long-term diuretic use, nor did any have a prior history of underlying kidney disease. Groups T and L did not differ significantly in terms of patient characteristics at the time of admission (*P* > 0.05) (Table 1). No patients had preoperative atrial fibrillation. No differences were seen in the general preoperative data, intraoperative conditions, duration of mechanical ventilation, length of ICU stay, the volume of blood transfused, or the length of catecholamine use between group T and the traditional diuretic group L (*p* > 0.05) (Table 1).

**Table 1:** Comparison of characteristics between groups L and T

Parameters	Group T (*n* = 21)	Group L (*n* = 24)	*P*
General			
Age (years)	60.52 ± 10.73	55.46 ± 14.66	0.199
Male (cases)	13 (61.9)	18 (75)	0.344
BSA	1.82 ± 0.18	1.84 ± 0.21	0.752
BMI	25.08 ± 2.65	25.43 ± 3.98	0.734
Smoke (cases)	12 (57.1)	14 (58.3)	0.936
Comorbidities			
Hypertension (cases)	16 (76.2)	17 (70.8)	0.685
Diabetes (cases)	2 (9.5)	4 (16.7)	0.670
Stroke (cases)	1 (4.8)	2 (8.3)	1.000
Coronary artery disease (cases)	0	2 (8.3)	0.491
Preoperative cardiac condition			
Acute myocardial infarction (cases)	0	1 (4.2)	1.000
Pericardial effusion (cases)	10 (47.6)	9 (37.5)	0.493
Moderate or above aortic regurgitation (cases)	4 (19)	4 (16.7)	1.000
Etiology			
Aortic arteriosclerosis (cases)	21 (100)	23 (95.2)	1.000
Marfan syndrome (cases)	0	1 (4.8)	1.000
Preoperative laboratory examinations			
ALT(U/L)	118.19 ± 26.78	40.08 ± 20.04	0.495
Albumin(g/L)	36.69 ± 5.38	39.97 ± 9.24	0.203
Creatinine(umol/L)	88.81 ± 39.16	73.83 ± 23.80	0.387
Urea nitrogen (mmol/L)	7.14 ± 2.56	6.76 ± 2.34	0.562

### Surgery information

All the patients received the intervention of the ascending aorta only or the Frozen Elephant Trunk Technique (FETT) with the concurrent intervention of the ascending aorta. Groups T and L did not differ significantly in terms of surgical methods, operative time, extracorporeal circulation time, the proportion of patients requiring DHCA, and the length of DHCA (all *P* > 0.05–) (see Table 2).

**Table 2:** Comparison of surgery between groups L and T

Parameters	Group T (n = 21)	Group L (n = 24)	*P*
Surgery information			0.124
Isolate ascending aorta replacement/Bentall Procedure (cases)	16	13	–
Ascending aorta replacement + FETT (min)	5	11	–
Surgery time (min)	323.57 ± 72.99	371.67 ± 103.78	0.083
Cardiopulmonary bypass time (min)	137.38 ± 30.85	146.67 ± 9.03	0.425
DHCA (cases)	8 (38.1)	14 (58.3)	0.175
DHCA time (min)	16.00 ± 6.78	17.69 ± 6.52	0.576

### Postoperative situation

Groups T and L did not differ significantly in terms of the duration of postoperative mechanical ventilation, time spent in ICU hospitalization, length of postoperative hospitalization, postoperative blood transfusion volume, time of use of catecholamines, and the amount of intravenous diuretic drugs administered (all *P* > 0.05).

All patients with POAF within 3–7 days after being transferred from ICU to the cardiac surgery ward, and were converted to sinus rhythm within 3 days after receiving symptomatic treatment such as amiodarone conversion.

The occurrence of POAF in group T was significantly less than that in group L (*P* = 0.023) (see Table 3 for details).

**Table 3:** Comparison in postoperative information between groups L and T

Parameters	Group T (n = 21)	Group L (n = 24)	*P*
Duration of mechanical ventilation (h)	38.67 ± 47.11	33.21 ± 38.35	0.741
Duration of ICU stay (days)	2.90 ± 2.66	2.42 ± 2.02	0.662
Catecholamine use (h)	35.71 ± 11.99	32.29 ± 5.78	0.148
Postoperative blood transfusion volume (ml)	723.81 ± 126.46	695.83 ± 80.45	0.727
Time from surgery to discharge (days)	18.81 ± 6.31	16.63 ± 6.21	0.209
Number of intravenous diuretic injections (times)	10.38 ± 2.17	6.75 ± 1.49	0.083
Complications			
POAF (cases)	3 (14.3)	11 (45.8)	0.023
Stroke (cases)	1 (4.8)	1 (4.2)	1
Gastrointestinal bleeding (cases)	1 (4.8)	0	1
Subarachnoid hemorrhage (cases)	1 (4.8)	0	1

**Fig. 1:**
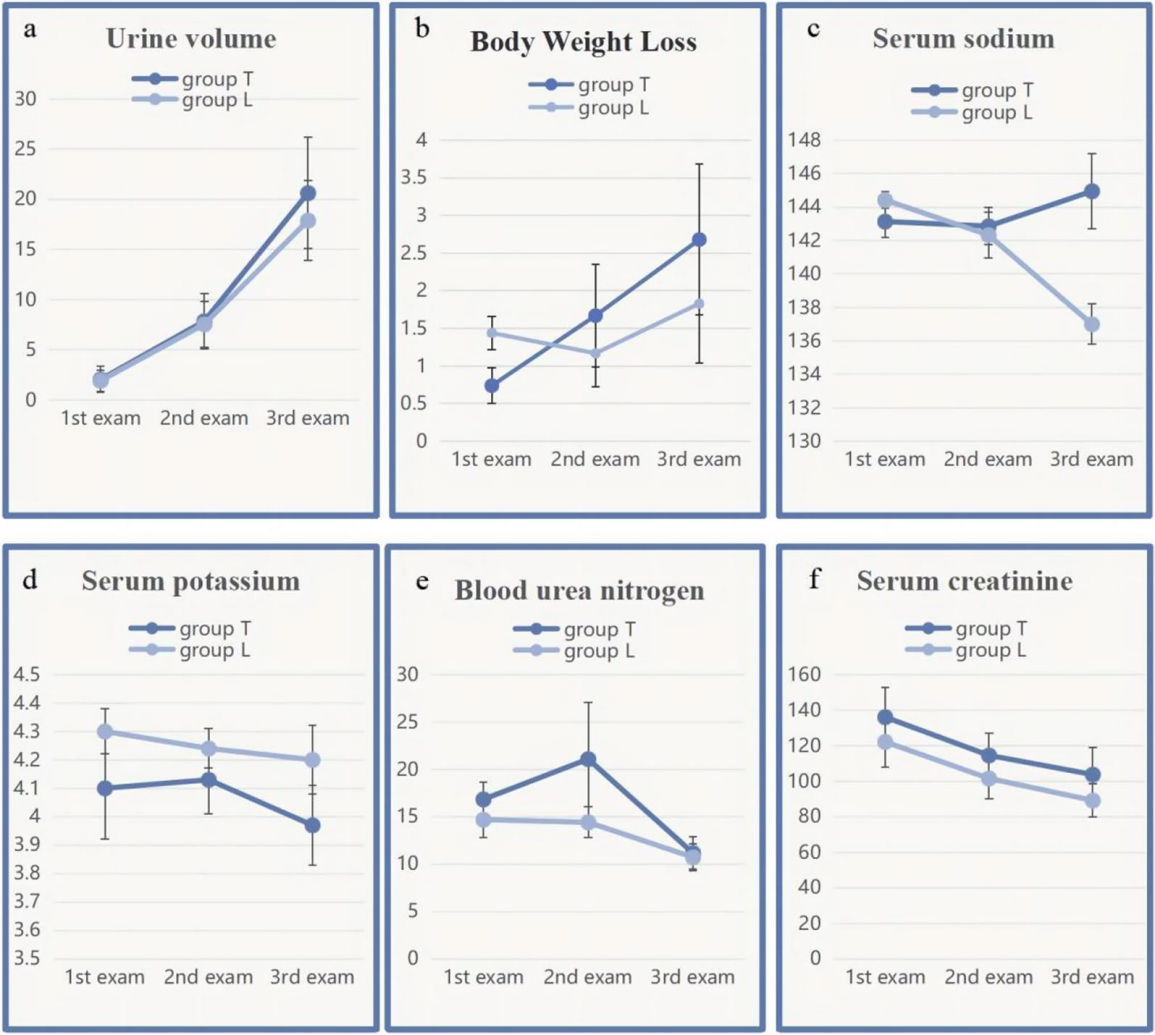
Changes in cumulative urine volume, body weight, and serum levels of sodium, potassium, creatinine, and urea nitrogen **a**–**f**: *Significant difference between groups T and L

**Fig. 2:**
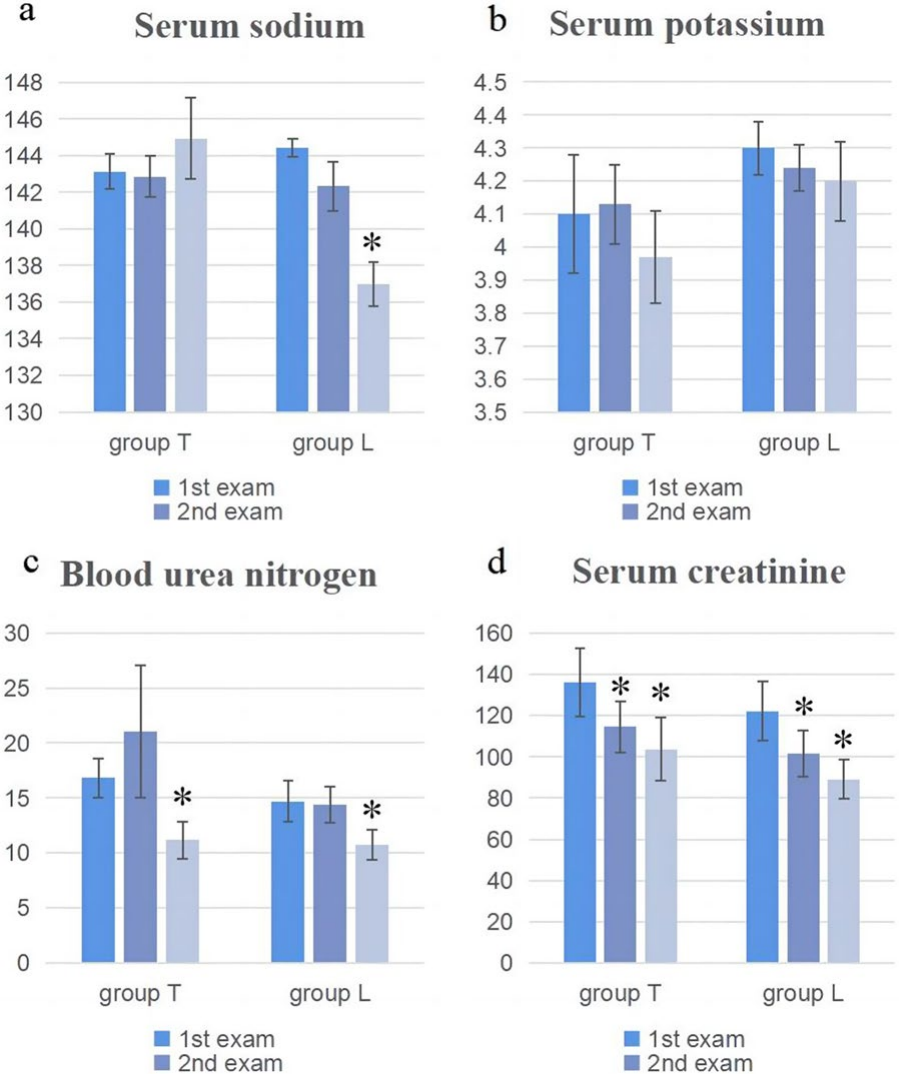
Changes in serum levels of sodium, potassium, creatinine, and urea nitrogen **a**–**d**: *Significantly different from day 1 measurement

After transfer from the ICU to the cardiovascular surgery ward, the urine volumes and change in body weight were slightly greater in group T than in group L but the differences were non-significant (*P* > 0.05). No changes were seen in serum potassium, creatinine, and urea nitrogen between the groups at any time (all *P* > 0.05). Although the sodium ion level did not differ between the groups on days 1 and 3 after the transfer, it was significantly elevated in group T on day 7 (*P* = 0.001). See Figs. 1, 2, and Table 4 for details.

**Table 4:** Changes in cumulative urine volume and levels of serum sodium, potassium, creatinine, and urea nitrogen

	Group T (n = 21)	Group L (n = 24)	*P*
Urine volume (L)			
Day 1	2.05 ± 1.28	1.92 ± 1.06	0.820
Day 3	7.86 ± 2.76	7.54 ± 2.30	0.613
Day 7	20.62 ± 5.56	17.88 ± 3.99	0.154
Loss of body weight (kg)			
Day 1	0.74 ± 0.24	1.44 ± 0.22	0.061
Day 3	1.67 ± 0.68	1.17 ± 0.44	0.554
Day 7	2.68 ± 1.00	1.83 ± 0.79	0.524
Serum sodium (mmol/L)			
Day 1	143.14 ± 0.97	144.42 ± 0.50	0.436
Day 3	142.86 ± 1.11	142.33 ± 1.36	0.784
Day 7	144.95 ± 2.24	137.58 ± 1.21	0.001
Serum potassium (mmol/L)			
Day 1	4.1 ± 0.18	4.30 ± 0.08	0.453
Day 3	4.13 ± 0.12	4.24 ± 0.07	0.250
Day 7	3.97 ± 0.14	4.20 ± 0.12	0.246
Serum creatinine (umol/L)			
Day 1	136.05 ± 16.75	122.13 ± 14.32	0.569
Day 3	114.52 ± 12.46	101.54 ± 11.37	0.439
Day 7	103.71 ± 15.10	89.13 ± 9.32	1.000
Blood urea nitrogen (mmol/L)			
Day 1	16.83 ± 1.77	14.68 ± 1.89	0.265
Day 3	21.08 ± 6.91	14.41 ± 1.64	0.682
Day 7	11.17 ± 1.69	10.72 ± 1.39	0.585

The serum sodium, potassium, creatinine, and urea nitrogen levels in the groups were compared with those measured at the time of the first examination. The serum sodium levels differed between days 1 and 7 after transfer from the ICU in group L (*P* = 0.001). The creatinine and urea nitrogen levels in both groups also differed significantly between days 1 and 7 (all *P* < 0.05). See Table 5 for details.

**Table 5:** Comparison of the day 3 and day 7 levels of serum sodium, potassium, creatinine, and urea nitrogen with the day 1 results

	Group T (n = 21)	Group L (n = 24)
	*P*	*P*
Serum sodium (mmol/L)		
Day 1 versus day 3	0.572	0.053
Day 1 versus day 7	0.681	0.001
Serum potassium (mmol/L)		
Day 1 versus day 3	0.149	0.637
Day 1 versus day 7	0.351	0.449
Serum creatinine (umol/L)		
Day 1 versus day 3	0.037	0.001
Day 1 versus day 7	0.006	0.001
Blood urea nitrogen (mmol/L)		
Day 1 versus day 3	0.498	0.415
Day 1 versus day 7	0.006	0.006

## Discussion

The present study compared the efficacy of tolvaptan and traditional diuretics after acute Stanford type A AD.

In previous post-cardiac surgery studies, the subjects were primarily patients after cardiac surgery. Most of these patients have basic heart failure before the operation, and most need cardiotomy and other heart-damaging operations during the process [1–8]. The object of this study was acute Stanford type A AD surgery. There was no heart failure before the procedure, and no cardiotomy was required during the procedure. This may also have a certain impact on the patient's postoperative fluid management.

In previous studies of tolvaptan in cardiac surgery, patients' postoperative urine output was mainly 10-15L/7 days [10, 17–19]. But in this study, patients had significantly more urine output. The patient's postoperative fluid management and changes in urine volume depend on the patient's postoperative intake and excretion adjustment. In this study, we applied diuretics to remove fluid retention while supplementing the patient's basic daily fluid requirements. This could explain why our patient had increased urine output following surgery. We noted the patient's weight changes to more accurately reflect the patient's fluid changes. It was observed that both tolvaptan and conventional diuretics achieved satisfactory voiding volume and body weight loss after surgery, and there was no difference in the need for intravenous diuretics.

Before surgery, there were no significant changes in serum creatinine and urea nitrogen levels between the two groups. This was related to the level of preoperative ischemia. Laboratory results depend heavily on the effect of AD on the blood supply to the organs [18]. All patients in this study were Penn's type Aa patients. Although the dissection was extensive in some patients, there was no adverse organ perfusion. This also allows us to bserve diuretics' effect on these patients better.

Previous reports have shown that tolvaptan increases urine output significantly more than conventional diuretics [10, 19]. The current study observed a slight, but non-significant, elevation in urine output in the T group than in the L group. And no significant difference was observed in weight loss between the T group and the L group. The long-term use of loop diuretics may result in diuretic resistance often caused by sodium reabsorption in the distal tubules. Tolvaptan can reduce sodium loss and thus mitigate diuretic resistance [20]. However, patients undergoing surgery for type A AD do not usually have histories of long-term loop diuretic use, and none of the present study participants had a history of traditional diuretic use. These patients would not be resistant to diuretics and, consequently, would likely be highly sensitive to loop diuretics [21].

Furthermore, Nishi et al. evaluated the response to tolvaptan in patients undergoing cardiovascular surgery. The amount of urine excreted after tolvaptan administration was discovered to be dependent on the degree of fluid retention before and during surgery, even though there was no substantial difference between responders and non-responders in terms of preoperative comorbidities or blood tests [22]. Patients with type A AD do not experience significant fluid retention before surgery as there is no underlying heart disease, which may render these patients unresponsive to tolvaptan. However, the sample size was relatively small and future studies are needed for confirmation.

Hypernatremia is defined as excessive sodium levels in the blood. It usually happens when a person has a low fluid intake or excessive fluid loss. Hypernatremia is an important public health issue. In this study, blood sodium levels in the T group were considerably higher after one week than in the L group.Significantly raised sodium levels were also seen in the L group on day 7 after transfer to the cardiac surgery ward compared to day 1. Studies have found that the development of hypernatremia was not dependent on the type of protocol used [23, 24]. It was found that although sodium levels increased over time in patients receiving tolvaptan, the rise was significantly lower than in patients not receiving tolvaptan [12, 24].

The serum potassium ion level is also a concern in diuretic patients. Here, no differences were seen in the potassium levels over time in the two groups, nor between the groups simultaneously. A previous meta-analysis of serum potassium in patients on tolvaptan concluded no difference in the mean serum potassium levels in patients with or without tolvaptan [25].

Tolvaptan has been reported to maintain renal blood flow during diuresis without activating the renin–angiotensin–aldosterone system [26, 27]. It has been suggested that using tolvaptan in cardiac surgery patients may enhance renal perfusion and stimulate the elimination of urea nitrogen [23, 28]. However, in this study, the levels of blood creatinine and urea nitrogen in both T and L groups showed a significant downward trend with the prolongation of postoperative time. Neither creatinine nor urea nitrogen levels differed between the groups at similar time points. Previous results showed no significant difference in renal function indicators, such as serum creatinine, between patients receiving loop diuretics and those receiving tolvaptan. None of the patients in this study had renal disease, and serum creatinine and urea nitrogen on a postoperative day 1 were only slightly elevated. It has been reported that the protective effect of tolvaptan on the kidney increases as essential renal function decreases [29]. The question arises as to whether this protective action of tolvaptan may not be as strong as expected in patients with type A AD. Nevertheless, tolvaptan does not have serious adverse effects on kidney function, at least compared with traditional diuretics.

Risk factors for POAF after conventional cardiac surgery include age and cardiac structural changes [30, 31]. Recent studies have shown that volume overload, electrolyte imbalance, acute kidney injury, and sympathetic nervous system activation are also risk factors for POAF after cardiac surgery [32]. Based on the patient's bodily fluid change, weight change, and electrolyte status, in conjunction with the patient's surgical circumstances. Additionally, we compared the surgical procedure, operation time, postoperative pain control, oxygen saturation level, and other parameters associated with postoperative new-onset atrial fibrillation. However, there were no statistically significant differences observed.

The current study observed significantly less development of new atrial fibrillation in the T group than in the L group. In all patients with new-onset AF, AF occurred 3 days after admission from the ICU to the cardiac surgery unit. Although the weight loss rate of group L appeared to be faster than that of group T on day 1, it was not statistically significant. Previous studies have shown no significant adverse effects of the drug on the occurrence of complications after thoracotomy [30, 31]. Recent studies have suggested that tolvaptan may reduce the occurrence of atrial fibrillation after cardiac surgery by inhibiting the activation of the renin–angiotensin–aldosterone axis and sympathetic nervous system [32].

A variety of factors influenced the duration of hospitalization. No significant length of hospital stay differences was seen between the two groups.

This study has several limitations. First, this was a single-center retrospective study with a limited number of patients that could cause selection bias and residual confounding. Second, the duration of medication was defined from the time of transfer from the ICU to the cardiovascular surgery ward, thus excluding the course of treatment in the ICU. Further limitations are that several important laboratory indicators such as urine ions and urine osmotic pressure were not included.

## Conclusion

In conclusion, both tolvaptan and traditional diuretics were found to be effective and safe for patients with acute Stanford type A AD. Tolvaptan may be associated with reducing the incidence of postoperative atrial fibrillation.

## Data Availability

The datasets generated and analyzed during the current study are available from the corresponding author upon reasonable request.

## Abbreviations

AD: Aortic dissection
ALT: Alanine transaminase
BMI: Body mass index
BSA: Body surface area
DHCA: Deep hypothermic circulatory arres
FETT: Frozen elephant trunk technique
ICU: Intensive care unit
POAF: Postoperative atrial fibrillation
